# Prolonged survival in a HER2 positive metastatic breast cancer patient with brain metastases treated with TDM1 after failure of next generation HER2 targeted therapies: case report

**DOI:** 10.3389/fonc.2026.1865921

**Published:** 2026-07-28

**Authors:** Benedetta Trevisan, Deborah Cosentini, Greta Schivardi, Francesca Piazza, Giulia Scartabellati, Laura Moretti, Lucia Vassalli, Giuseppe Ippolito, Francesco Donato, Alfredo Berruti, Marta Laganà, Rebecca Pedersini

**Affiliations:** 1 Department of Medical and Surgical Specialties, Radiological Sciences and Public Health, Medical Oncology, University of Brescia, Aziendo Socio-Sanitaria Territoriale (ASST) Spedali Civili, Brescia, Italy; 2 Department of Medical and Surgical Specialties, Radiological Sciences and Public Health, Unit of Hygiene, Epidemiology, and Public Health, University of Brescia, Aziendo Socio-Sanitaria Territoriale (ASST) Spedali Civili, Brescia, Italy; 3 Struttura Semplice Dipartimentale (SSD) Breast Unit, Aziendo Socio-Sanitaria Territoriale (ASST) Spedali Civili of Brescia, Brescia, Italy

**Keywords:** brain metastasis, breast cancer, case report, HER-2, Treatment strategy

## Abstract

**Background:**

Brain metastases (BM) occur in up to 50% of patients with HER2-positive metastatic breast cancer (MBC) and represent a major clinical challenge due to limited CNS drug penetration. In recent years, novel anti-HER2 agents—such as trastuzumab deruxtecan (T-DXd) and tucatinib—have shown promising intracranial efficacy and are now recommended after progression on first-line therapies. However, no prospective data are available to guide optimal treatment sequencing, particularly in the CNS setting.

**Case presentation:**

We report the case of a 37-year-old woman with HER2-positive, hormone receptor-negative MBC and symptomatic BM who progressed after treatment with T-DXd and a tucatinib-based regimen. Remarkably, fourth-line therapy with Trastuzumab Emtansine (T-DM1) resulted in a durable intracranial response, maintained over 24 months, with good clinical tolerance and manageable side effects.

**Results:**

this case underscores the potential of T-DM1 to remain an effective treatment option even after the failure of next-generation HER2-targeted therapies. The sustained response may be attributed to tumor biology, prior radiotherapy-induced modulation of the blood–brain barrier, or enhanced drug delivery to CNS lesions. Given its favorable toxicity profile and ease of administration, T-DM1 retains clinical relevance in selected patients.

**Conclusion:**

T-DM1 may offer meaningful benefit in HER2+ MBC with BM, especially where newer agents fail or are contraindicated. Further studies are needed to clarify optimal sequencing strategies.

## Introduction

Human epidermal growth factor receptor 2–positive (HER2+) breast cancer (BC) accounts for approximately 15–20% of all BC cases and is associated with an aggressive phenotype higher recurrence risk and poorer prognosis (1). Brain metastases (BM) are more frequent in HER2+ BC (25–50%) (2). Anti-HER2 therapies, starting with trastuzumab, significantly improved progression-free (PFS) and overall survival (OS) mainly through extracranial disease control, but benefits are less evident in patients with BM. Management of BM often includes local therapies like surgical resection, stereotactic radiosurgery, stereotactic radiotherapy, or whole-brain radiation (3).

Novel systemic agents, including antibody-drug conjugates (ADCs) and target therapies have demonstrated promising efficacy of Her2+ MBC who have progressed on at least one prior HER2-directed therapy (4). Several studies have demonstrated the intracranial activity of T-DXd in patients with HER2+ MBC with BM. Pooled analysis of the DESTINY-Breast 01/02/03 trials confirmed robust CNS efficacy of T-DXd, even in heavily pretreated patients (5), while DESTINY-Breast 12, specifically designed to assess CNS activity, validated these findings (6). HER2-CLIMB study showed that the tucatinib-based combination provided a significant survival benefit in patients with HER+ MBC, including those with active or stable brain metastases previously treated with trastuzumab, pertuzumab, and T-DM1 (7, 8). Based on these results, T-DXd and tucatinib are recommended therapeutic options for patients with HER2+ MBC, according to both ASCO and ESMO clinical guidelines, following progression on first-line treatment with pertuzumab and trastuzumab even in the presence of BM (3, 9).

Despite the increasing availability of anti-HER2 agents, prospective data guiding the optimal sequencing of these therapies remains lacking especially in the setting of CNS progression.

Here, we report the clinical case of a patient with HER2+ MBC and BM who experienced rapid disease progression on next-generation HER2-targeted therapies, yet achieved an unexpectedly durable clinical and radiological response with T-DM1—raising important questions about therapeutic sequencing and resistance mechanisms.

## Case presentation

**Figure 1:**
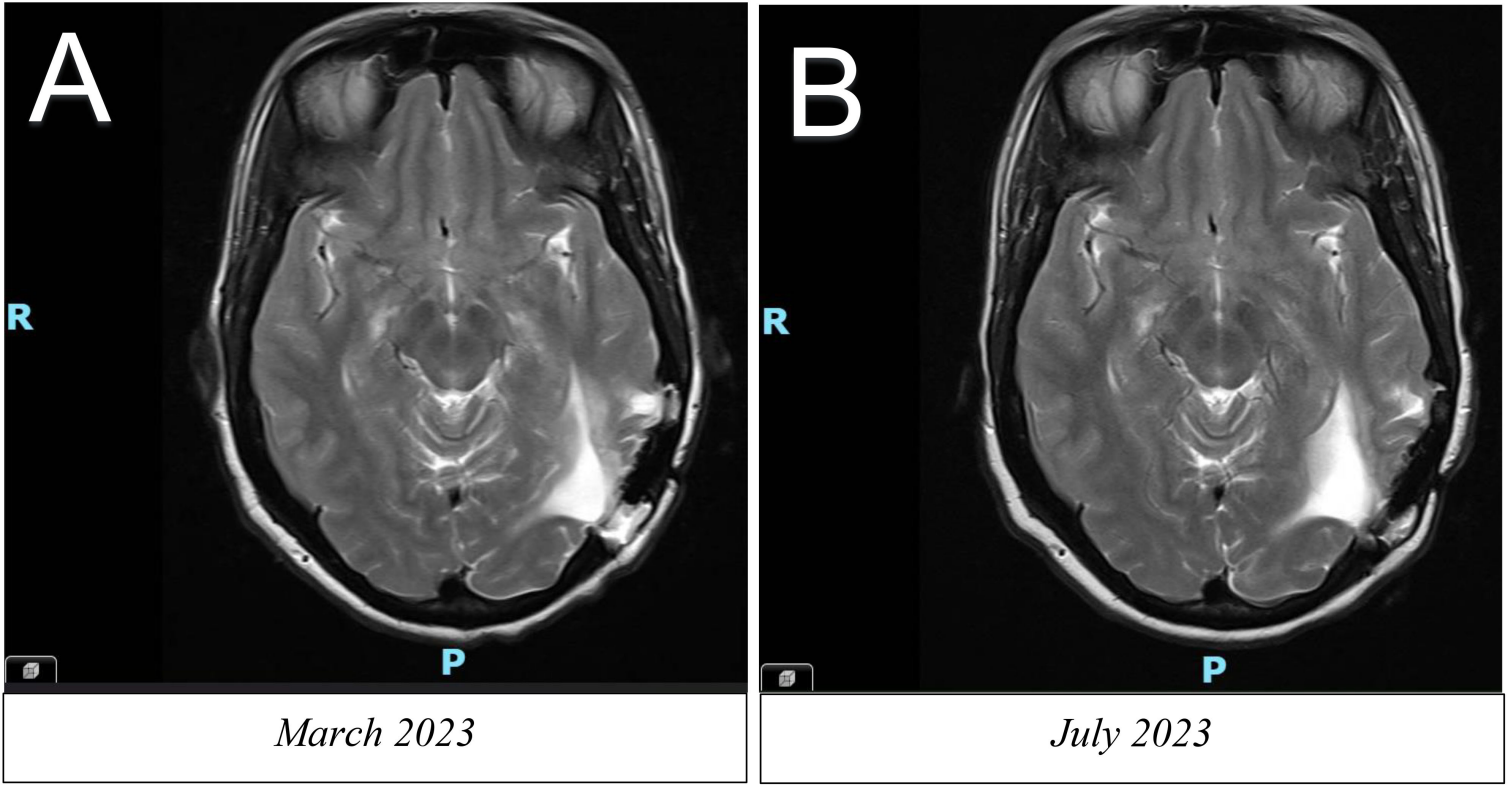
Brain MRI performed in March 2023 at the initiation of T-DM1 therapy **(A)** and after four months of TDM1 treatment **(B)**.

**Figure 2:**
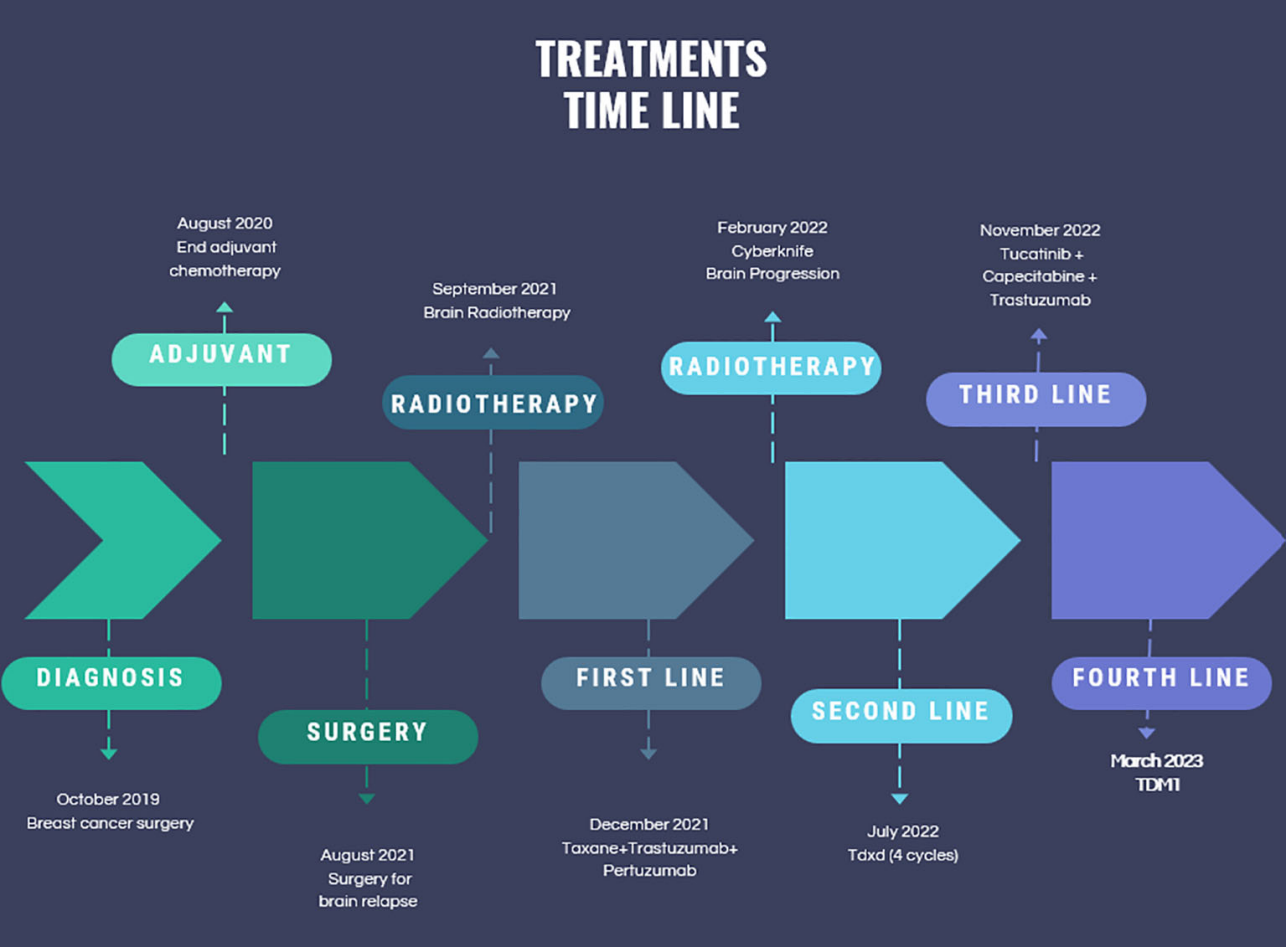
Patient Treatments Time Line.

In March 2019, a 37-year-old black woman was diagnosed with HER2+, hormone receptor-negative locally advanced BC. The patient had no family history of breast cancer. Her medical history was otherwise unremarkable, with no significant comorbidities. She had two full-term pregnancies and had used oral contraceptives for one year. A core needle biopsy of a 6 cm retroareolar lesion in the left breast revealed an invasive carcinoma, and fine-needle aspiration cytology confirmed axillary lymph node involvement. According to immunohistochemical analysis, the tumor was negative for estrogen and progesterone receptors, exhibited a high proliferative activity with a Ki-67 index of 38%, and showed HER2 overexpression with an immunohistochemical score of 3 +. Given the young age of the patient, comprehensive genetic testing was also performed, with no germline mutations detected. Following multidisciplinary discussion, and considering the disease stage, tumor biology, and the patient’s excellent clinical condition (ECOG Performance Status 0), neoadjuvant systemic therapy with docetaxel, pertuzumab, and trastuzumab was started in April 2019 (The taxane dose was reduced to 80% of the original dosage from the eighth cycle due to grade 2 transaminase elevation and grade 2 asthenia). The patient underwent a mastectomy with axillary lymph node dissection in October 2019 due to the favorable clinical response; histopathological examination revealed a complete pathological response in the breast (ypTis) and no residual axillary disease. Adjuvant therapy with trastuzumab and pertuzumab was continued for 14 additional administrations, completing the planned one-year treatment duration, and was discontinued in August 2020. Twelve months after discontinuation of anti-HER2 therapy, in August 2021, the patient reported a new-onset headache that was unresponsive to analgesics. Contrast-enhanced brain computed tomography (CT) and magnetic resonance imaging (MRI) revealed a single enhancing intra-axial lesion in the left posterior temporal lobe, associated with significant perilesional edema, while chest and abdomen CT scan showed no evidence of disease. Following multidisciplinary evaluation, the patient underwent surgical resection of the intracranial lesion, and histopathological analysis confirmed that the MBC was consistent with the molecular profile of the primary tumor. In September 2021, a follow-up brain MRI revealed disease recurrence at the surgical site, and the patient subsequently underwent stereotactic body radiotherapy (SBRT) with a dose of 24 Gy to the posterior temporal lesion. Considering the patient’s oncological history, the technical challenges associated with performing a brain biopsy in a previously treated area, and the well-established propensity of HER2-positive breast cancer to metastasize to the brain, first-line systemic treatment with paclitaxel, trastuzumab, and pertuzumab was initiated; however, in December 2021, after only three months of treatment, MRI revealed disease progression with two new lesions: an 8 mm lesion in the temporo-occipital region and a 6 mm lesion herniating through the posterior craniotomy site. Due to the oligoprogressive nature and accessibility of the anatomical site, the patient was treated with cyberknife radiosurgery in February 2022 and continued with the same systemic therapy. By July 2022, new lesions were detected in the surgical cavity, left cerebellum, and left occipital lobe, accompanied by the onset of headache and visual disturbances. Given the prior radiation exposure, additional radiotherapy was not indicated, and in August 2022, the patient commenced second-line therapy with T-DXd (4 cycles, 5.4 mg/kg, neutropenia grade 3 after the third cycle and nausea grade 1 were reported). After three months of treatment, in November 2022, brain MRI demonstrated further disease progression. Then was introduced third-line treatment with tucatinib (300 mg orally twice daily) in combination with trastuzumab (8 mg/kg intravenous loading dose followed by 6 mg/kg every 21 days) and capecitabine (1,000 mg/m² orally twice daily on days 1–14 of each 21-day cycle), which was well tolerated but resulted in a progression-free survival of only four months. In March 2023, brain MRI (**Figure 1A**) revealed further intracranial progression (while no new extracranial lesions appeared) in the left occipital and cerebellar regions, with cortico-meningeal enhancement in the left cerebellum leading to the initiation of fourth-line treatment with T-DM1(3.6 mg/kg intravenous every 21 days). At the first radiological re-evaluation after four months of T-DM1 treatment, performed in July 2023, brain MRI demonstrated an initial intracranial response, with resolution of cortico-meningeal enhancement in the left cerebellum and a reduction in contrast enhancement in the left temporo-occipital region, as it shown in **Figure 1B**. The intracranial response has been maintained across all subsequent follow-up imaging assessments, including the most recent in March 2026. Thirty-six months after treatment initiation, the patient remains on T-DM1 therapy with good clinical tolerance, experiencing only mild fatigue and loss of appetite in the days following treatment administration. The patient’s clinical history is resumed in **Figure 2**.

## Discussion

Despite the detrimental impact of CNS metastases on quality of life (QoL), therapeutic strategies for advanced or recurrent HER2+ BC are evolving rapidly, driven by the emergence of novel anti-HER2 agents, including ADCs and tyrosine kinase inhibitors. In particular, T-DXd and tucatinib-based regimens have shown unprecedented intracranial efficacy and are now widely recommended in updated clinical guidelines (10, 11).

HER2CLIMB-02 study showed that tucatinib-based regimen improves PFS even in patients with BM (7.8 vs 5.7 months; HR 0.64), suggesting that adding tucatinib to T-DM1 could be a clinically meaningful option for selected patients (12).

However, the expanding landscape of HER2-targeted therapies has not been matched by prospective clinical trials addressing treatment sequencing, especially after the failure of next-generation agents. A critical point highlighted by this case is that the patient received T-DXd before the tucatinib-based regimen. This sequence (T-DXd then tucatinib-based therapy) was not typical for the HER2CLIMB (10) trial’s enrollment, as T-DXd gained prominence and approval later. This patient’s progression on T-DXd prior to a tucatinib-based regimen underscores the growing challenge of managing patients who have exhausted newer, highly effective agents. The subsequent remarkable response to T-DM1 in this context further emphasizes the need for data on optimal sequencing in heavily pretreated settings, particularly after T-DXd failure.

In the absence of robust sequencing data, clinicians are often left to make empiric decisions beyond the third line of therapy, particularly in patients with CNS involvement.

Prior to the approval of T-DXd, T-DM1 was the standard second-line therapy based on the results of the EMILIA study (13–15) and it is now recommended when T-DXd is contraindicated, such as in patients with interstitial lung disease (9).

Although T-DM1 is currently considered a therapeutic option after newer anti-HER2 agents in clinical guidelines (3, 9), the sustained response of our patient highlights its continued relevance in selected cases and raises important questions about the mechanisms underlying this response. This observation also prompts a broader discussion on the potential benefits of introducing T-DM1 earlier in the treatment algorithm for selected patients with BM. Although its impact on PFS is limited, T-DM1 has demonstrated improved OS and clinical benefit in patients with BM HER2+ MBC, as shown in EMILIA (13–15), TH3RESA (16), and KAMILLA (17) studies, with additional support from case reports and retrospective analyses highlighting neurological symptom improvements (18–21).

In KATHERINE, adjuvant T-DM1 significantly reduced invasive disease recurrence or death compared with trastuzumab in patients with residual HER2+ disease after neoadjuvant therapy. However, no reduction in the incidence of brain metastases was observed (22). This suggests that T-DM1 ability control micrometastatic CNS disease may be limited. By contrast, durable intracranial responses reported in the metastatic setting may reflect differential activity in established brain lesions, particularly where the blood–tumor barrier is disrupted. In the absence of prospective sequencing data T-DM1 as fourth-line therapy after T-DXd and tucatinib can be rationalized by its favorable safety profile and by reintroducing an antibody–drug conjugate following failure of a small-molecule HER2-directed regimen, while maintaining systemic and intracranial activity. In this patient with HER2+ MBC and symptomatic brain metastases, T-DM1 achieved a sustained intracranial response lasting over 24 months, despite rapid disease progression on both T-DXd and a tucatinib-based combination.

This exceptional response might be explained by individual tumor biology, radiotherapy-induced disruption of the blood–tumor barrier (BTB), or a potential synergistic effect that enhanced T-DM1 penetration and activity within the CNS. Several studies have demonstrated that T-DM1 is capable of penetrating the altered blood–tumor barrier (BTB), and emerging evidence suggests that prior radiotherapy may further enhance this permeability (23). In our patient, radiotherapy-induced changes in the BTB may have facilitated T-DM1 delivery to the CNS, potentially amplifying its therapeutic effect and contributing to the sustained clinical and radiological response observed. Given that T-DXd and T-DM1 have similar sizes (10–15 nm) and were both administered after radiotherapy, we cannot suggest a greater penetration into the brain for T-DM1. However, we cannot rule out that the molecule administered later may have encountered a more compromised blood–brain barrier in this specific case (24). We acknowledge that, although plausible, the proposed mechanisms remain hypothetical and cannot be validated based on a single observation. It cannot be excluded, although in the absence of strong and concrete supporting data, that interposing two ADCs with a drug having a different mechanism of action, such as a small molecule targeted therapy, may have enhanced the activity of T-DXd. Since the efficacy of second-generation ADCs, such as T-DM1, is strongly associated with target expression, it is possible that tucatinib treatment may have influenced HER2 receptor re-expression, or that the drug’s efficacy could be partially mediated by HER2-independent mechanisms (25).

This case underscores several key points: 1-T-DM1 may retain therapeutic efficacy even after failure of next-generation HER2-targeted agents; 2-prior radiotherapy may enhance ADC activity in the CNS by increasing BTB permeability (the hypothesis that radiotherapy can disrupt the blood–tumor barrier could enhance intracranial drug delivery, though at the cost of potential toxicities such as radionecrosis); 3-in the absence of sequencing data, T-DM1 remains a viable and clinically valuable option beyond the third line, particularly in patients with good performance status and limited brain disease; 4-T-DM1 offers a favorable safety profile, ease of administration, and quality-of-life preservation, which are especially relevant in the context of CNS disease. Nevertheless, the findings of this report should be interpreted with caution given the inherent limitations of a single-case report. The observed clinical benefit cannot be generalized to the broader population, and additional evidence is required before the proposed mechanism, including enhanced blood–brain barrier penetration, can be considered definitive.

The potential role of additional treatment lines in a therapeutic landscape where T-DM1 has been partially replaced by more effective agents may still be relevant in selected young patients, such as the one described in this case report. The patient’s emotional burden of managing metastatic breast cancer with brain involvement was compounded by her young age and the demands of raising a small child. Despite the challenges of rapid disease progression through multiple treatment lines, during clinical encounters the patient consistently expressed her willingness to pursue active treatment and her desire to maximize the time spent with her family while maintaining the best possible quality of life.

In conclusion, this case strongly underscores the potential efficacy of T-DM1 in the treatment of BM HER2+ MBC, even after failure of next-generation HER2-targeted agents. The patient achieved a remarkable and sustained intracranial response, maintained for over 24 months, with excellent clinical tolerance and preserved QoL. This outcome is particularly significant given the rapid progression observed under more recent therapies, and it highlights the continued therapeutic value of T-DM1 in a clinical context where CNS-active options remain limited. Although currently positioned after newer agents in clinical guidelines, T-DM1 has demonstrated durable intracranial activity and could be considered in selected patients, particularly those with favorable clinical conditions, limited brain disease, or contraindications to next-generation therapies. Although speculative, prior radiotherapy and specific tumor biology may have contributed to enhanced drug penetration across the blood–tumor barrier, further supporting its efficacy in the CNS.

The lack of prospective data on treatment sequencing beyond the third line of therapy makes this case particularly relevant in clinical practice. This case challenges current sequencing paradigms and supports the reconsideration of T-DM1 as an active agent with durable CNS efficacy, highlighting the need for prospective studies or real-world evidence on optimal sequencing in HER2+ MBC with BM.

## Data Availability

The original contributions presented in the study are included in the article/Supplementary Material. Further inquiries can be directed to the corresponding author.
